# Realized genomic selection across generations in a reciprocal recurrent selection breeding program of *Eucalyptus* hybrids

**DOI:** 10.3389/fpls.2023.1252504

**Published:** 2023-10-27

**Authors:** Guilherme Ferreira Simiqueli, Rafael Tassinari Resende, Elizabete Keiko Takahashi, João Edesio de Sousa, Dario Grattapaglia

**Affiliations:** ^1^ Plant Genetics Laboratory, EMBRAPA Genetic Resources and Biotechnology, Brasilia, Brazil; ^2^ School of Agronomy, Federal University of Goiás (UFG), Goiânia, GO, Brazil; ^3^ Department of Forestry, University of Brasília (UnB), Brasília, DF, Brazil; ^4^ CENIBRA Celulose Nipo Brasileira SA, Belo Oriente, MG, Brazil

**Keywords:** genomic selection, GBLUP, HBLUP, growth, hybrid tree breeding, SNPs, eucalyptus

## Abstract

**Introduction:**

Genomic selection (GS) experiments in forest trees have largely reported estimates of predictive abilities from cross-validation among individuals in the same breeding generation. In such conditions, no effects of recombination, selection, drift, and environmental changes are accounted for. Here, we assessed the effectively realized predictive ability (RPA) for volume growth at harvest age by GS across generations in an operational reciprocal recurrent selection (RRS) program of hybrid *Eucalyptus*.

**Methods:**

Genomic best linear unbiased prediction with additive (GBLUP_G), additive plus dominance (GBLUP_G+D), and additive single-step (HBLUP) models were trained with different combinations of growth data of hybrids and pure species individuals (*N* = 17,462) of the G_1_ generation, 1,944 of which were genotyped with ~16,000 SNPs from SNP arrays. The hybrid G_2_ progeny trial (HPT267) was the GS target, with 1,400 selection candidates, 197 of which were genotyped still at the seedling stage, and genomically predicted for their breeding and genotypic values at the operational harvest age (6 years). Seedlings were then grown to harvest and measured, and their pedigree-based breeding and genotypic values were compared to their originally predicted genomic counterparts.

**Results:**

Genomic RPAs ≥0.80 were obtained as the genetic relatedness between G_1_ and G_2_ increased, especially when the direct parents of selection candidates were used in training. GBLUP_G+D reached RPAs ≥0.70 only when hybrid or pure species data of G_1_ were included in training. HBLUP was only marginally better than GBLUP. Correlations ≥0.80 were obtained between pedigree and genomic individual ranks. Rank coincidence of the top 2.5% selections was the highest for GBLUP_G (45% to 60%) compared to GBLUP_G+D. To advance the pure species RRS populations, GS models were best when trained on pure species than hybrid data, and HBLUP yielded ~20% higher predictive abilities than GBLUP, but was not better than ABLUP for ungenotyped trees.

**Discussion:**

We demonstrate that genomic data effectively enable accurate ranking of eucalypt hybrid seedlings for their yet-to-be observed volume growth at harvest age. Our results support a two-stage GS approach involving family selection by average genomic breeding value, followed by within-top-families individual GS, significantly increasing selection intensity, optimizing genotyping costs, and accelerating RRS breeding.

## 1 Introduction

The concept of using the “total allelic” (Nejati-Javaremi et al., 1997) or “total genomic” (Haley and Visscher, 1998) relationship from marker data to derive estimates of breeding values, subsequently termed “genomic selection” (GS) (Meuwissen et al., 2001), has revolutionized animal and plant breeding in the last 20 years. This paradigm shift in thinking on how to use DNA marker data to increase the rate of genetic gain per unit time was made possible by the convergence of the time-proven quantitative genetics framework and novel high-throughput genomic technologies to interrogate thousands of genome-wide single-nucleotide polymorphisms (SNPs) (Grattapaglia et al., 2018). In essence, GS is a biometric method to predict the genetic merit of genotyped but yet-to-be-phenotyped individuals or families, called selection candidates, based on prediction equations built from a population genetically related to these selection candidates, called training population, for which both phenotypes and genotypes are available. GS has become the routine breeding strategy for a number of animal breeding programs (Van Eenennaam et al., 2014), and its rapid adoption is taking place in plant breeding as well (Crossa et al., 2017; Hickey et al., 2017), while new methods are constantly developed to improve the nonstop challenge of complex trait prediction (Ahmadi and Bartholomé, 2022).

In forest tree breeding, the contemplation of GS started with deterministic simulations some 12 years ago (reviewed in Grattapaglia, 2022). Predictions were made for major enhancements in the rate of genetic gain per unit time, by radically reducing generation interval, increasing selection intensity, and improving the accuracy of breeding values. GS uses a marker-based kinship matrix (G) among the genotyped individuals instead of the standard pedigree-based matrix (A). The G matrix accounts for the Mendelian segregation among family members as well as any ancestral cryptic relationships existing among individuals within and across generations, unknown by the contemporary pedigree. As Ignacy Misztal straightforwardly put it, *“think of genomic selection as the animal model with more accurate relationships”* (Misztal, 2011). Several reviews have now been published, compiling results of experimental studies and describing the fundamental and practical aspects of GS applied to tree breeding with an emphasis on the mainstream plantation species (Grattapaglia, 2014; Isik, 2014; Grattapaglia, 2017; Grattapaglia et al., 2018; Lebedev et al., 2020; Ahmar et al., 2021; Isik, 2022). Overall, experimental reports have been positive, predicting advantages over conventional pedigree-based selection irrespective of species and deployment strategy whether by improved seedling or selected individual clones. Gains would derive from accelerated breeding through the possibility of much earlier selection together with increased selection intensity, especially for late expressing and/or low heritability traits. Furthermore, a consensus has been reached that the foundational population used to train the genomic prediction model must represent the relevant genetic diversity to the breeding program and be closely related to the selection candidates (Grattapaglia, 2022; Isik, 2022).

Forest tree breeding programs, however, usually involve large populations with hundreds of individuals and families, typically operating on tight budgets. Although genotyping has become more affordable, implementation of GS by collecting biological samples and genotyping all individual trees is frequently logistically and financially not possible. An alternative is the single-step genomic-based BLUP (GBLUP) approach (Legarra et al., 2009), a.k.a. HBLUP, by which a blended relationship H matrix consolidates the pedigree information (A matrix) of many more non-genotyped individuals and the genomic relationship (G matrix) of a smaller set of genotyped individuals. The added information of the genotyped individuals is propagated to all trees with this combined approach, providing genetic relationship connections across offspring and parents, frequently resulting in more reliable breeding values of ungenotyped trees when compared with the pedigree-based ABLUP. Additionally, the inclusion of phenotypic information of ungenotyped trees in the HBLUP model may lead to an increment in predictive ability of the genotyped trees when compared to the standard GBLUP (Cappa et al., 2017; Ratcliffe et al., 2017; Cappa et al., 2019; Paludeto et al., 2021; Callister et al., 2022; Cappa et al., 2022; Walker et al., 2022).

Species of *Eucalyptus* have been one of the main workhorses of GS experimental work toward breeding applications in forest trees (reviewed in Lebedev et al., 2020). Nine species in the subgenus *Symphyomyrtus*, among more than 700 species in the genus, make up over 95% of the world’s eucalypt plantations (Harwood, 2011), providing fast volume growth, broad adaptability, and multipurpose wood (Grattapaglia et al., 2012). The vast genetic diversity found within species and the possibilities to exploit complementarity and heterosis of contrasting gene pools into eucalypt hybrids deployed by clonal propagation have been the major drivers of genetic gains in tropical regions. The extensively planted “urograndis” hybrid (*E. urophylla* × *E. grandis*) developed in the 1980s in Brazil (Brandão et al., 1984; Eldridge et al., 1993) is the most paradigmatic example, currently representing the benchmark for clonal forest productivity in tropical regions and the world. It combines the fast growth of *E. grandis* with the increased tolerance to biotic and abiotic stresses and better rooting ability of *E. urophylla*, supplying wood suitable to different industrial applications. Besides being the pillar of eucalypt plantations in Brazil (Lima et al., 2019), “urograndis” hybrids have been bred and planted in Congo (Bouvet et al., 2009b), South Africa (Retief and Stanger, 2009; Van Den Berg et al., 2018), and Southern China (Wu et al., 2011) and have shown promising results in the Southern United States (Kellison et al., 2013).

The success of the “urograndis” hybrid has fueled reciprocal recurrent selection (RRS) programs between *E. grandis* and *E. urophylla* in Brazil, Congo, and South Africa to exploit the significant contribution of dominance variance detected in a number of studies (Bouvet et al., 2009b; Retief and Stanger, 2009; Van Den Berg et al., 2018; Lima et al., 2019). In the typical RRS strategy, pure species trees of *E. grandis* and *E. urophylla* are intermated to generate hybrid half- or full-sib families deployed in terminal hybrid progeny trials where top individuals are selected, further tested as clones, and eventually recommended for commercial propagation. In the standard RRS, genetically superior pure species trees of each species are backward selected based on their performance as parents of hybrids, and then intermated to establish pure species progeny trials where the best trees will be selected to form the improved reciprocal populations for the next breeding cycle.

The RRS strategy is a two-step breeding cycle that takes considerable time because the advancement of the pure species reciprocal populations depends on the hybrid progeny trial data for backwards selection, although a forward selection variant has been proposed (Nikles, 1992) and evaluated by simulations as more efficient (Kerr et al., 2004). In RRS, GS would therefore have a two-pronged positive impact to significantly accelerate breeding cycles for “urograndis” hybrids. First, GS would be applied to the hybrid offspring of terminal crosses at the seedling stage for individual tree selection, precluding the progeny trial, allowing moving directly to field validation clonal trials of genomically selected seedlings. Second, GS applied to the pure species progeny trials, followed by accelerated flowering by top grafting, would shorten the time needed to advance the two reciprocal breeding fronts. Simulation studies in bovine (Mcewin et al., 2021), oil palm (Cros et al., 2015), and wheat (Rembe et al., 2019) have indicated that the incorporation of GS in RRS would be a valuable method to shorten generation intervals and improve long-term gains.

While GS across breeding generations is already standard commercial practice in domestic animals (Van Eenennaam et al., 2014) and annual crops (Albrecht et al., 2014; Michel et al., 2016; Osorio et al., 2021), very scant experimental data exist in forest trees. In trees, genomic predictive abilities have largely been reported as projected estimates based on cross-validated data in the same generation, and not as effectively accomplished predictive abilities across a parent–offspring generational gap at the operational harvest age of the genomically selected trees. The long time necessary to reach the final operational harvest age and match it to what the genomic data had predicted at the seedling stage has been the main obstacle to accomplish GS studies across generations in forest trees. The topic has been approached mostly through “back-tested” retrospective studies, taking advantage of existing sequential breeding generations (Bartholome et al., 2016; Isik et al., 2016; Haristoy et al., 2023) or prospective studies where only juvenile growth could be assessed (Thistlethwaite et al., 2019).

In this work, we evaluated the effectively realized predictive ability (RPA) for volume growth at harvest age by forward GS across generations in a eucalypt hybrid progeny trial. To ensure merging of data from different experiments deployed at different times, we applied age adjustment models on prior generation data used for model training. Additive only, additive plus dominance, and single-step genomic models were used for predictions. For the production component of the RRS program, the RPA was evaluated for selecting individuals and families in a terminal “urograndis” hybrid progeny trial. The ranks of the estimated breeding (EBVs) or genotypic values (EGVs) for volume growth at harvest age were matched to their genomically predicted counterparts (GEBVs and GEGVs) estimated when they were seedlings. For the breeding component of the RRS program, we estimated the predictive abilities (PA) by cross-validation for the reciprocal pure species advancement, assessing the different contributions of pure species or hybrid data to train prediction models.

## 2 Materials and methods

### 2.1 Experimental populations

**Figure 1:**
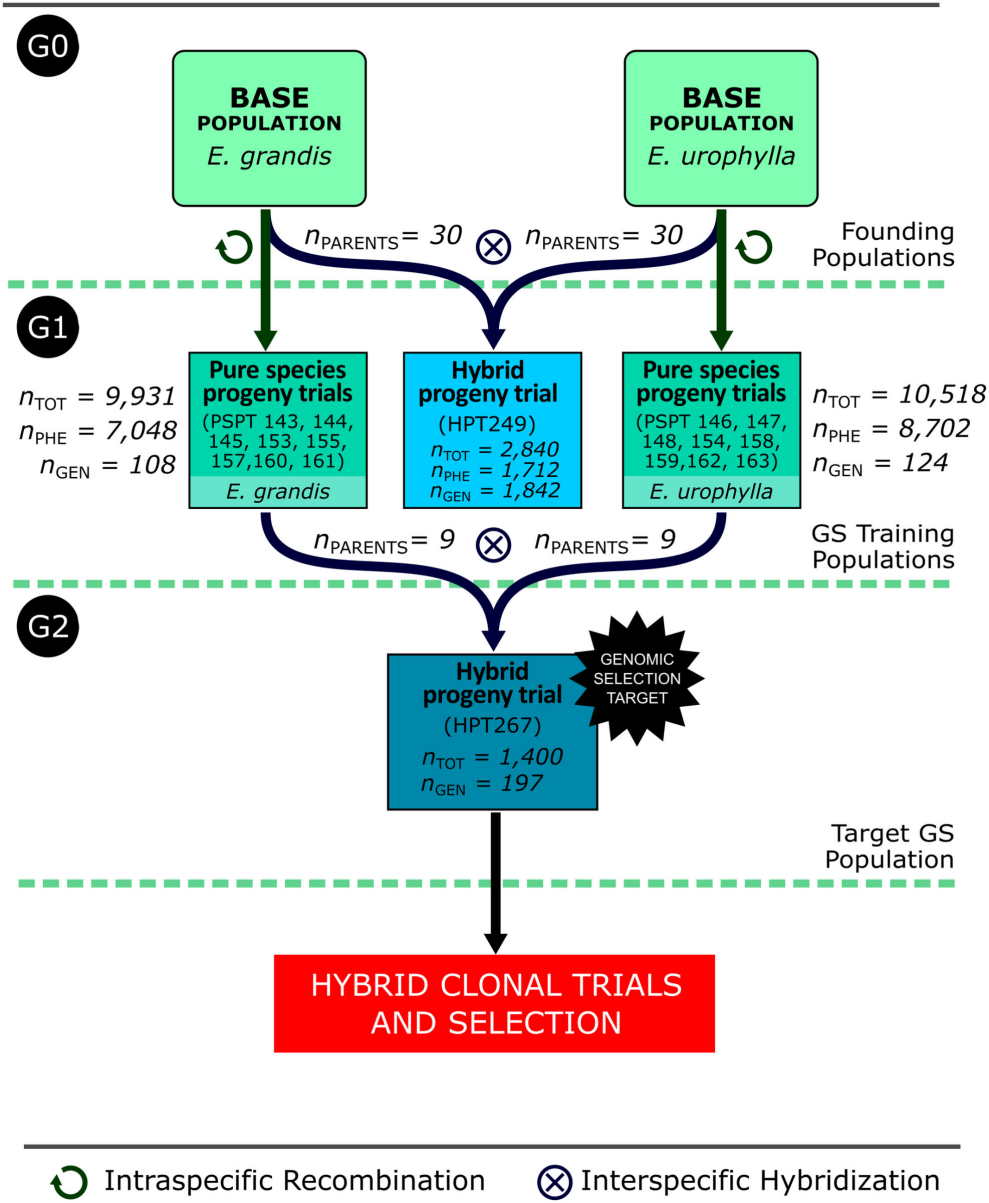
Summary flowchart of the breeding populations involved in the forward GS experiment indicating the generations to which they belong (G_0_, G_1_, and G_2_), the total number of individual trees in each trial (n_TOT_), the number of trees phenotyped (n_PHE_), and the number of trees genotyped (n_GEN_). The total number of individuals in G_1_ used in model training is the sum of the phenotyped individuals (7,048 + 1,712 + 8,702 = 17,462). PSPT: Pure Species Progeny Trial; HPT: Hybrid Progeny Trial.

The study was carried out with operational breeding populations of the RRS breeding program of Cenibra S.A. in Brazil (**Figure 1**). The program was originally started with 30 pure species breeding parents for each species, *E. grandis* and *E. urophylla*, here called G_0_ generation. These trees were selected between 2004 and 2007 based on their performance as parents of “urograndis” hybrids in diverse hybrid progeny trials evaluated between 1985 and 2005. For each one of the two species separately, the 30 elite trees were recombined using a polymix design with a mixture of pollen collected from all 30 parents, generating 30 intraspecific half-sib families for each species. Between 2008 and 2009, these half-sib families were tested in 16 pure species progeny trials (PSPT), 8 for each species. The trials were established in a randomized complete block design with 36–50 blocks and 30 families planted as single tree plots plus four or five commercial clones as checks. In total, these PSPT ultimately provided data for 9,931 trees for *E. grandis* and 10,518 for *E. urophylla* as part of the G_1_ breeding population for this work. Between 2010 and 2012, half-sib hybrid families were generated again by using a polymix design of the 30 G_0_ *E. grandis* parents pollinating the 30 G_0_ *E. urophylla* parents and *vice versa*. These 60 hybrid families derived from the top G_0_ parents plus checks were established in the G_1_ hybrid progeny trial HPT249 in a randomized complete block design with 50 replications in single tree plots in April 2013. For this HPT249, 1,712 G_1_ trees were measured and used in this work to train prediction models.

In 2015, the G_1_ PSPTs were subject to between- and within-family selection, and 30 breeding parents were selected for each species. Using 9 of the 30 parents for each species that flowered synchronously, the G_2_ hybrid progeny trial HPT267 was created and deployed in 2016 to serve as a terminal trial for individual tree selection. Selected hybrid individuals would not be used as parents for the next generation, but subsequently clonally propagated and tested as clones towards final commercial recommendation. The HPT267 was established in randomized complete block design with 35 full-sib families, 28 unique and 7 reciprocal, with 40 replications in single tree plots, totaling 1,400 trees tested plus three commercial checks. Prior to the establishment of the field trial of HPT267, a sample of 200 seedlings out of the 1,400 were randomly sampled in 34 of the 35 families, covering 27 unique families, for SNP genotyping and genomic prediction (**Figure 1**). Three plants failed genotyping and 197 were therefore used in the study. Their genomic predicted breeding and genotypic values for volume growth would later be compared to their breeding and genotypic values estimated from their volume growth measured at harvest age (6 years).

Diameter at breast height (DBH) and tree height (HT) were measured at around ages 3 and 6 years in all the trials described above. Wood volume (VOL) in cubic meters was estimated using a taper factor of 0.45. Mean annual increment (MAI) in m^3^ ha^−1^ year^−1^ was calculated by multiplying the total tree volume by 1,200 trees per hectare and dividing the result by the average years of growth. In summary, the GS experiment across generations involved the G_1_ generation trials, HPT249 and all PSPTs employed individually or combined as training sets, to predict the G_2_ HPT267 as the GS target trial.

### 2.2 Genotypic data

SNP genotype data were obtained with the EuCHIP60K chip (Illumina, Inc.) (Silva-Junior et al., 2015) or with the Axiom 72K Eucalyptus array (ThermoFisher, Santa Clara, CA). The following sets of G_1_ trees were genotyped and used either individually or combined as training sets for genomic prediction of individuals of the G2 target population HPT267: (1) 1,842 F_1_ individuals of HPT249; (2) the 18 parents of progenies in HPT267, 9 of *E. grandis* and 9 of *E. urophylla*; and (3) 99 *E. grandis* and 115 *E. urophylla* trees randomly sampled in the same G_1_ PSPTs where the 18 parents of HPT267 were selected (**Figure 1**). The GS candidates of generation G_2_ were the 1,400 trees of HPT267. For this target population, only 197 trees were genotyped. Genomic DNA was extracted from leaf tissue samples of these trees using an optimized Sorbitol+CTAB method for high-quality DNA from wood samples (Inglis et al., 2018), quantified with a Nanodrop 2000 spectrometer and adjusted to concentrations between 20 and 40 ng μL^−1^. Only SNPs with call rate ≥0.90 and minor allele frequency (MAF) ≥0.01 and common to the two SNP genotyping arrays were used. All SNPs were of the type A/G, A/C, T/G, or T/C. SNP data were exported either from GenomeStudio 2.0 or from Axiom Analysis Suite 4.0.1 formatted as AA, AB, and BB where the A allele is the SNP nucleotide base A or T and the B allele is nucleotide base C or G at the SNP. Missing data were imputed based on the expected value $2p_{i}$ of the B allele frequency $\left(p_{i}\right)$ according to the following rule: imputed to homozygote AA when $2p_{i}<2/3$ ; imputed to homozygote BB when $2p_{i}\geq 4/3$ ; and imputed to heterozygote AB when $\;2/3\leq 2p_{i}<4/3$ . Genotypes AA, AB, and BB were then converted to 0, 1, and 2, respectively, for the subsequent analyses. Using the SNP data, pedigree checking was carried out for all genotyped full-sib individuals of HPT249 and no errors were found. For the 232 pure species individuals of G_1_ the SNP data were consistent with their half-sibship status, but no maternal G_0_ genotypes were available for full pedigree verification.

### 2.3 Age data adjustment

The different G_1_ trials had been measured at slightly different ages, around age 2.76 (2.64 to 2.98 years) and 5.40 (4.94 to 6.14 years). To allow data consolidation to equal ages, linear and non-linear models were fitted to the raw volume growth data to adjust the age differences of the trees across the experimental trials to the same two ages points, 2.76 and 5.40 years, hereafter called ages 3 and 6 years for brevity, corresponding to the arithmetic mean of the measurement ages of all trials. Different random regression adjustments were evaluated: a linear model (Eq. 1) and three non-linear models: Logistic 1 (Eq. 2), Logistic 2 (sigmoid) (Eq. 3), and the Gompertz curve (Gompertz, 1833) (Eq. 4) as follows:


(1) $$y_{i}=\;\beta_{0_{i}}+\beta_{1_{i}}x$$

(2) $$y_{i}=\;\frac{1}{1+\beta_{0_{i}}e^{\beta_{1_{i}}x}}$$

(3) $$y_{i}=\;\frac{1}{1+{\left(x/\beta_{0_{i}}\right)}^{\beta_{1_{i}}}}$$

(4) $$y_{i}=\;e^{-e^{\beta_{0_{i}}-\beta_{1_{i}}x}}$$

where $y_{i}$ is the adjusted volume of the *i*th tree, $\beta_{0_{i}}$ e $\beta_{1_{i}}$ are the parameters of the respective models, and x is the age. MAI was subsequently obtained by dividing the total volume ( $m^{3}\;{\text{h}}^{a-1}$ ) by the age. The nonlinear models were linearized (**File S1**) and the regressions were adjusted using these models for each individual tree using the lme4 package (Bates et al., 2015) in the R software (R Core Team, 2022). Illustrative examples of the fitting of the different models are provided (**Figure S1**). Altogether, in addition to the unadjusted raw dataset, four different datasets were generated, containing measurement at two ages each.

### 2.4 Pedigree based BLUP (ABLUP) analyses

ABLUP estimated EBVs or EGVs were used as pseudo phenotypes in genomic predictions. EBVs and EGVs for the G_1_ and G_2_ individuals were obtained for the two measurement ages using two alternative univariate individual tree mixed models, one additive (model A; Eq. 5) and one additive-dominance (model A+D; Eq. 6):


(5) y= Xβ+Wα+Za+e

(6) y= Xβ+Wα+Za+Qd+e

where y is the vector of age-adjusted phenotypic values; β is the vector of fixed trial effects; α is the random block effect within trials, following $\alpha \;∼\;N\left(0,\sigma_{b}^{2}\;I\right)$ , where I is a diagonal matrix and $\sigma_{b}^{2}$ is the variance of block effects; a is the random additive genetic effect, following $a\;∼\;N\left(0,\sigma_{a}^{2}\;A\right)$ , where A is the average numerator Wright’s relationship matrix and $\sigma_{a}^{2}$ is the additive genetic variance; d is the vector of random dominance effects following $d\;∼\;N\left(0,\;\sigma_{d}^{2}\;D\right)$ , where D is the average dominance relationship matrix and $\sigma_{d}^{2}$ is variance of dominance effects; e is the vector of the random residual effect following $e\;∼\;N\left(0,\;I\sigma_{e}^{2}\right)$ , where $\sigma_{e}^{2}$ is the residual variance. *X*, *W*, *Z*, and *Q* are the incidence matrices relating fixed and random effects in the model to the measurements in vector y . The D matrix was estimated with the package nadiv (Wolak, 2012) in R (R Core Team, 2022) and the models were fitted with breedR (Munoz and Sanchez, 2014). The variance for residual and block effects was therefore considered homogeneous across the different trials in an attempt to fit a more parsimonious model. Nevertheless, the same models in Eqs. 5 and 6 were also fitted considering the possibility of residual variance heterogeneity across trials (Smith et al., 2001). Heterogenous variance models were fitted using ASReml R (version 4.1.0.176) for residual ( e ∼ N(0, R) ) and blocks ( α ∼ N(0,B) ), where $R\;={⊕}_{i=1}^{T}\sigma_{e_{i}}^{2}I_{n_{i}}$ , $B\;={⊕}_{i=1}^{T}\sigma_{b_{i}}^{2}I_{b_{i}}$ , $\sigma_{b_{i}}^{2}$ and $\sigma_{e_{i}}^{2}$ are the variance of blocks and residual variance in the *i*th trial, respectively, and ⊕ is the direct sum. Narrow- and broad-sense heritabilities were estimated, depending on the model, for each database for both measurements ages.

### 2.5 Comparison between EBVs and de-regressed EBVs

To evaluate whether the use of de-regressed EBVs as pseudo phenotypes would result in appreciably different results for our experimental data, de-regressed estimated breeding values (dEBVs) were also estimated for our training datasets by $EBV_{i}/r_{i}^{2}$ , where $r_{i}^{2}$ is the squared accuracy (reliability) of the *i*th individual (Garrick et al., 2009). Parental average effects were not removed, however, as it is well documented that removing relatedness between training sets and selection candidates radically reduces predictive abilities (Isik, 2022). To assess the relative effect of de-regression, correlations between EBVs and dEBVs were estimated for all individuals in all trials. Additionally, to evaluate whether the use of dEBVs instead of EBVs would impact the realized predictive abilities, the dataset for a specific case (Logistic 1 age adjusted; age 6; HBLUP) was implemented with dEBVs and results were compared to those obtained using EBVs.

### 2.6 Genomic-based BLUP analyses

Genomic evaluations were carried out using genomic best linear unbiased prediction (GBLUP) in the Sommer R package (Covarrubias-Pazaran, 2016) with two genomic models, additive (GBLUP_G; Eq. 7) and additive-dominance (GBLUP_G+D; Eq. 8) as follows:


(7) y= Xβ+Za+e

(8) y= Xβ+Za+Qd+e

Equations 7 and 8 were used to predict the individual tree GEBVs and GEGVs, respectively, where y is the vector of EBVs or EGVs (from Eqs. 5 and 6, respectively); β is the vector of fixed effects given by the overall mean; a is the random additive genomic effect in Eq. 7 and the genomic estimate of additive biological effect in Eq. 8, following a ~ $N\left(0,\sigma_{a}^{2}\;G_{A}\right)$ , where $G_{A}\;$ is the additive genomic relationship matrix; d is the vector of random dominance genomic effects following d ~ $\;N\left(0,\;\sigma_{d}^{2}G_{D}\right)$ , where $G_{D}$ is the dominance genomic relationship matrix and e is the vector of the random residual effect. *X*, *Z*, and *Q* are the incidence matrices relating fixed and random effects in the model to the measurements in vector y . The $G_{A}$ matrix was estimated by WW'/M (Yang et al., 2010), where W is the incidence matrix of the number of SNPs per standardized locus and *M* is the total number of SNPs, with MAF ≥ 0.01. The $G_{D}$ matrix (Vitezica et al., 2013) was estimated by the AGHmatrix package (Amadeu et al., 2016) in R, given by $KK'/\left[2\sum_{i=1}^{M}p_{i}q_{i}\left(1-2p_{i}q_{i}\right)\right]$ , where $K\subset \;\left\{0-2p_{i}q_{i};1-2p_{i}q_{i};0-2p_{i}q_{i}\right\}$ with homozygous genotypes coded as 0 and heterozygous genotypes coded as 1; $p_{i}$ is the major allele frequency of the *i*th SNP; $\;q_{i}=1-p_{i}$ ; and M is the total number of SNPs.

### 2.7 Single-step genomic BLUP analysis

Single-step genomic BLUP (HBLUP) was fitted in the Sommer R package with the following additive model (Eq. 9):


(9) y= Xβ+Za+e

where y is the vector of EBVs (from Eq. 5); β is the vector of fixed effects given by the overall mean; a is the random additive genomic effect, following a ~ $\;N\left(0,\sigma_{a}^{2}\;H_{A}\right)$ , where $H_{A}\;$ is the hybrid single-step relationship matrix that includes the expected relationships ( A ) from pedigree and the realized genomic relationships ( $G_{A}$ ) and e is the vector of the random residual effect. *X* and *Z* are the incidence matrices relating fixed and random effects in the model to the measurements in vector y . The $H_{A}$ matrix (Legarra et al., 2009) was obtained by:


$$H_{A}=\left[\begin{array}{cc} A_{11}+A_{12}A_{22}^{-1}\left(G_{A}-A_{22}\right)A_{22}^{-1}A_{21} & A_{12}A_{22}^{-1}G_{A}\\ G_{A}A_{22}^{-1}A_{21} & G_{A} \end{array}\right]$$

where $A_{11}$ corresponds to the expected additive relationships between the ungenotyped trees; $A_{12}$ and $A_{21}$ correspond to the expected additive relationships between the ungenotyped and genotyped trees and *vice versa*; and $A_{22}$ correspond to the expected additive relationships between the genotyped trees. The $H_{A}$ matrix was estimated with the AGHmatrix package (Amadeu et al., 2016) in R. The inverse of $H_{A}$ (Aguilar et al., 2010; Christensen and Lund, 2010) was estimated via:


$$H_{A}^{-1}=A^{-1}+\left[\begin{array}{cc} 0 & 0\\ 0 & G_{A}^{-1}-A_{22}^{-1} \end{array}\right]$$

$A^{-1}$ is inverse of Wright’s relationships matrix, $G_{A}^{-1}$ and $A_{22}^{-1}$ are the inverses of genomic and expected relationship matrices for genotyped individuals. A scaling factor ( τ ) of one was applied to $\tau \left(G_{A}^{-1}-A_{22}^{-1}\right)$ to capture all genomic information on the prediction of future genotypes (Aguilar et al., 2010) and $G_{A}$ was not scaled towards to $A_{22}$ (Christensen et al., 2012). Given the structure and variety of the existing relationships between the different G_1_ training sets and G_2_ selection candidates, we chose a parameter equal to 1 to use the total contribution of the genomic relationships to the prediction of selection candidates. The choice was also made in light of earlier results with similar eucalypt data showing only slight differences on the variance components when the tuning parameter decreased from 1.0 to 0.0 (Cappa et al., 2017).

### 2.8 Genomic prediction across generations

Genomic predictions in the G_2_ generation hybrid progeny trial HPT267 were carried out using the three genomic models (GBLUP_G, GBLUP_G+D, and HBLUP), trained with different combinations of the prior generation data as follows: (1) the preceding G_1_ hybrid generation represented by the HPT249, henceforth called HYBRIDS with 2,840 individuals, 1,842 of which were genotyped; (2) the nine *E. grandis* and nine *E. urophylla* G_1_ genotyped parents mated to create the HPT267, henceforth called PARENTS; (3) the preceding G_1_ generation pure species progeny trials, henceforth called UNCLES, which included phenotype data for 5,143 individuals of *E. grandis* and 5,190 individuals of *E. urophylla*, out of which genotype data were obtained for 108 and 124 trees for the two species, respectively; (4) the combined HYBRIDS+PARENTS dataset; (5) the combined HYBRIDS+UNCLES dataset; (6) the combined PARENTS+UNCLES dataset; and (7) the combined HYBRIDS+PARENTS+UNCLES dataset. The analysis therefore encompassed five phenotypic datasets (one unadjusted and four age-adjusted datasets); two individual tree genetic models (A and A+D); three genomic models (GBLUP_G, GBLUP_G+D and HBLUP); and seven training sets for each of two measurement age, totaling 420 analytical strategies.

All analytical strategies were evaluated for their genomic RPA for MAI in the target HPT267 hybrid generation G_2_. The RPAs were calculated by the Pearson correlation between the individuals’ GEBVs and their EBVs for the GBLUP_G and HBLUP models, or their GEGVs and EGVs for the GBLUP_G+D model. For the GBLUP models, the RPAs were estimated for the 197 genotyped individuals. HBLUP predictions were carried out for the 197 genotyped individuals to see whether the inclusion of phenotypic data of ungenotyped trees in training would improve predictions over GBLUP. GEBVs for the 1,203 ungenotyped individuals were also estimated by HBLUP with the inclusion of different types of G_1_ genotyped individuals in training and compared with the EBVs. Besides the estimates at the individual tree level, estimates of RPA at the family level were also calculated based on the family average EBVs or GEBVs for the 27 sampled families. Bias of the RPAs was estimated as 1−*b* where *b* is the slope of the regression line between EBVs and GEBVs or EGVs and GEGVs. A slope of *b*>1 indicates underestimation of the RPA, and *b*<1 denotes overestimation.

### 2.9 Rank correlations of ABLUP vs. GBLUP/HBLUP

The individual MAI tree ranks based on their GEBVs or GEGVs were compared to their ranks based on their EBVs or EGVs from ABLUP, respectively. The relationship between these two ordinal variables was evaluated by a Spearman rank correlation. The two ranks were subsequently used to calculate the coincidence rate (%) between the number of G_2_ selection candidate trees that would be genomically selected at the seedling stage and the number of trees that would be selected at harvest age based on their EBVs or EGVs for different proportions selected (2.5% to 50% selected).

### 2.10 Genomic predictions in the pure species populations

Genomic prediction models for each of the two pure species breeding populations of the RRS program (**Figure 1**) were built and cross-validated to be used for the forward selection of the pure species, *E. urophylla* and *E. grandis*, breeding cycles. Prediction models were trained for each species separately using either (1) only the pure species data from the respective species PSPTs, (2) only the preceding hybrid generation HPT249 data, or (3) the combined HPT249 and PSPT data (**Figure 1**). GBLUP and HBLUP were implemented under an additive model (Eqs. 8 and 10, respectively). Predictive abilities were estimated by 10-fold cross-validation when the training sets included the pure species data, and with direct validation when only data of the HPT249 were used for training because training and testing were different populations (hybrid vs. pure species). Predictive abilities were estimated by the mean of 10 Pearson linear correlations between the GEBVs and the respective validation sets. When only data of the HPT249 were used for training, a single Pearson correlation value was calculated. We estimated the predictive abilities yielded by ABLUP, GBLUP, and HBLUP to compare (1) the performance of GBLUP vs. ABLUP to predict genotyped but unphenotyped trees; (2) the performance of HBLUP vs. GBLUP to predict genotyped trees by including data of phenotyped but ungenotyped trees in the training set; and (3) the performance of HBLUP vs. ABLUP to predict ungenotyped trees by including data of genotyped trees in the training set.

## 3 Results

### 3.1 SNP genotyping data

Raw SNP data genotyped with the EuCHIP60K chip (Illumina, Inc.) (Silva-Junior et al., 2015) were exported from GenomeStudio 2.0 with GenCall > 0.15 and submitted to additional quality controls. Only SNPs that passed the following Illumina-recommended multi-variable metrics criteria were retained: (i) genotype clusters separation > 0.30; (ii) mean normalized intensity (*R*) value of the heterozygote cluster >0.2; (iii) mean normalized theta of the heterozygote cluster between 0.20 and 0.80; and (iv) >99% reproducibility across the replicated samples and >99% correct inheritance between generations. Additionally, only SNPs with call frequency ≥90% and MAF ≥ 0.01 for samples with call rate ≥85% were retained for further analyses. Out of the 60,904 unique SNPs present on the chip, data for 28,521 SNPs were ultimately retained after quality control. The 232 trees of the PSPTs were genotyped with the Axiom Euc72K Eucalyptus array (ThermoFisher, Santa Clara, CA) (https://www.thermofisher.com/order/catalog/product/br/en/551134) (D. Grattapaglia and O.B. Silva-Junior, unpublished), a second-generation *Eucalyptus* SNP platform improved over the EuCHIP60K chip that includes 67,683 autosomic *Eucalyptus* specific SNPs, 28,177 of which were selected from the previous EucHIP60k for quality and polymorphism across several *Eucalyptus* species. SNP data were exported using the Axiom Analysis Suite 3.1, using a dish quality control (DQC) value >0.82 and call rate CR >0.97 following the recommended “Best Practices Workflow” (Thermofisher, 2017). A total of 49,473 SNPs were classified as “Polymorphic High Resolution” with MAF > 0.01. Following consolidation of the datasets obtained with the two platforms for the 28,177 shared SNPs, a maximum of 16,861 and a minimum of 16,018 SNPs were used in the analyses, depending on the training set.

### 3.2 Age adjustment, heritabilities, and EBV/EGV estimation

Age adjustment resulted in slight reductions of residual variances at both ages (**Table S1**). Nevertheless, reductions were at most in the range of 2.3% to 2.5% at age 3, and between 1.8 to 2.1% at age 6. The Logistic 1 model was the most efficient in reducing the residual variance. Results hereafter are presented mainly for this age-adjusted dataset. Narrow-sense ABLUP estimated heritabilities for all G_1_ and G_2_ trials combined were h_a_^2^= 0.240 and 0.210, and broad-sense heritabilities were slightly higher at h_g_^2 ^= 0.250 and 0.220 at ages 3 and 6, respectively. The slight improvement of age adjustment was also reflected in the estimates of narrow-sense (h_a_^2^) and broad-sense (h_g_^2^) heritabilities for all G_1_ and G_2_ trials combined (**Table S1**). Narrow-sense ABLUP heritabilities for MAI were also estimated specifically in the G_2_ trial HPT267, under a purely additive model at h_a_^2 ^= 0.59 and 0.53 at ages 3 and 6, respectively (**Table 1**). By including dominance, the narrow-sense heritabilities decreased to h_a_^2 ^= 0.43 and 0.40, while broad-sense heritabilities were higher than narrow-sense ones, estimated at h_g_^2 ^= 0.60 and 0.54. When SNP data for 197 trees were included in the hybrid relationship matrix (HBLUP), heritabilities decreased to 0.45 at age 3, and more pronouncedly to 0.23 at age 6. Narrow- and broad-sense genomic heritabilities were estimated for the HPT267 under the three genomic models, using the seven G_1_ training sets. Genomic heritabilities, both narrow and broad sense, estimated using training data were considerably higher than the standard heritabilities estimated using the pedigree or hybrid matrix for the target trial, reaching values close to unity when PARENTS and UNCLES were included in training and when the HBLUP model was used. Under the GBLUP_G+D model, broad-sense genomic heritabilities were higher than narrow sense by 20% to 35% (**Table S2**).

**Table 1:** Heritabilities ( $h_{a}^{2}\;$ narrow sense; $h_{g}^{2}$ broad sense) for MAI (mean annual increment) in wood volume in the G_2_ trial HPT267 using age-adjusted data (Logistic 1 model), using ABLUP pedigree relationship matrices, A = additive; A+D = additive + dominance; and the HBLUP hybrid matrix H that included SNP data for 197 genotyped individuals of the 1,400 in the trial.

Fitted ABLUP model	Age	*N*	Additive variance	Dominance variance	Residual variance	$h_{a}^{2}$	$h_{g}^{2}$
A	3	1,400	305.16	–	211.18	0.59	–
H	3	1,400	232.49	–	279.42	0.45	–
A+D	3	1,400	207.31	81.90	193.16	0.43	0.60
A	6	1,400	403.71	–	356.03	0.53	–
H	6	1,400	157.79	–	517.88	0.23	–
A+D	6	1,400	285.87	103.15	330.70	0.40	0.54

No substantial difference was seen between the estimates of EBVs/EGVs when using a model assuming homogeneous experimental residual variances across trials versus a model with heterogenous variances. The correlation between the EBVs/EGVs estimated with a homogeneous versus a heterogenous variance model was, on average, 0.978 ± 0.019, median 0.984 for all trials, and all correlations were above 0.887 (**Table S3**). The additive-dominance model (Eq. 6) with heterogenous variances only converged for the age-unadjusted data at age 6. These results supported a more parsimonious homogeneous residual variances model for our experimental data for estimating the EBVs/EGVs. Furthermore, applying de-regression to the EBVs did not result in appreciably different values for our experimental data. High correlations were seen between dEBVs and EBVs for all training sets. The correlations were, on average, 0.979 ± 0.014, median 0.978, minimum value 0.930 (**Table S4**). Therefore, all genomic prediction results in this study are based on using standard EBVs or EGVs as pseudo phenotypes.

### 3.3 Realized genomic predictions in the G_2_ hybrid trial

**Figure 2:**
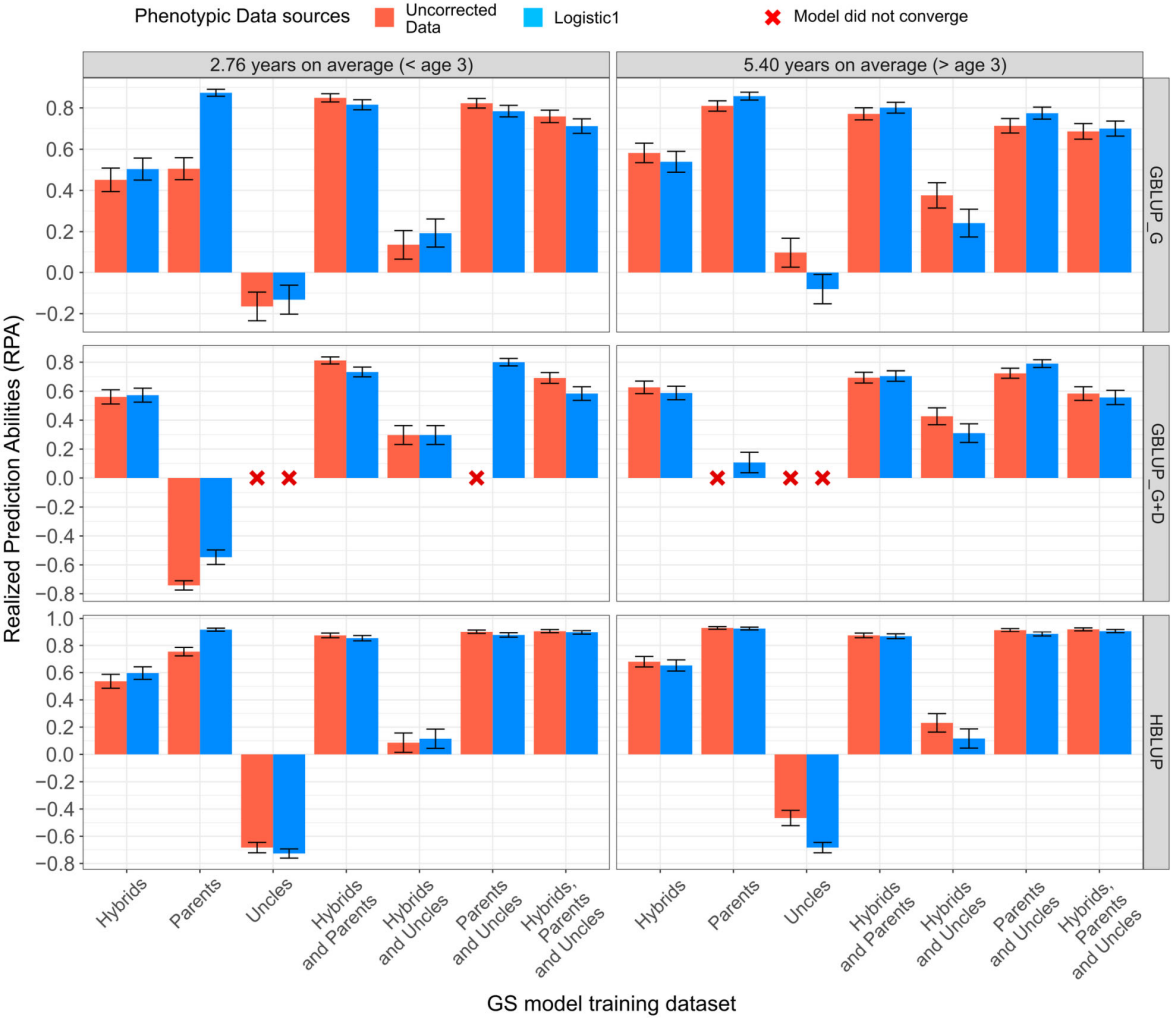
Realized genomic predictive abilities for mean annual increment (MAI) in tree growth at ages 3 and 6 years, estimated with an additive (GBLUP_G), an additive + dominance (GBLUP_G+D) model, and an additive HBLUP model, trained with different training datasets.

RPAs in the G_2_ trial HPT267 showed a substantial difference depending on what prior G_1_ generation training set was used (**Figure 2**; **Table 2**). For brevity, only the RPAs obtained with the age-unadjusted and Logistic 1 model adjusted data for age 6 are shown (**Figure 2**), and summarized together with other GS performance parameters (**Table 2**), but the results for all analytical strategies are provided (**Table S5**). In general, RPAs for the age-adjusted and -unadjusted data were largely equivalent with a few cases where differences were observed depending on the training set, indicating that age adjustment could be useful but, by and large, the impact on the final predictions was minimal. Estimated RPAs for age 3 and 6 were similar (**Figure 2**).

**Table 2:** Summary of the genomic selection results in the G_2_ generation (HPT267) for mean annual increment (MAI) in tree volume at age 6, using Logistic 1 age adjustment for the different combinations of genomic models and training sets.

Genomic model	Training set*	# Individuals in the training set	# Individuals predicted in HPT267	RPA	Bias (1−*b*)	Spearman’s rank correlation	Coincidence rate of selected trees			
							2.5%	5%	10%	20%
GBLUP-G	H	1,842	197	0.539	−0.988	0.530	0.00	20.00	25.00	60.00
	P	18	197	0.857	−0.126	0.870	60.00	60.00	40.00	70.00
	U	214	197	−0.081	1.274	−0.047	0.00	0.00	0.00	7.50
	H + P	1,860	197	0.801	−0.877	0.795	40.00	50.00	40.00	57.50
	H + U	2,056	197	0.241	0.199	0.282	0.00	0.00	15.00	37.50
	P + U	232	197	0.775	−1.008	0.784	20.00	30.00	40.00	65.00
	H + P + U	2,074	197	0.700	−0.723	0.717	0.00	0.00	30.00	60.00
GBLUP-G+D	H	1,842	197	0.588	−0.950	0.575	0.00	0.00	25.00	60.00
	P	18	197	0.107	0.648	0.475	0.00	0.00	5.00	32.50
	U	214	197	NA	NA	NA	0.00	20.00	30.00	52.50
	H + P	1,860	197	0.705	−0.811	0.694	0.00	0.00	20.00	37.50
	H + U	2,056	197	0.310	0.022	0.318	20.00	20.00	40.00	52.50
	P + U	232	197	0.790	−0.776	0.797	0.00	0.00	20.00	47.50
	H + P + U	2,074	197	0.557	−0.456	0.559	0.00	0.00	25.00	60.00
HBLUP 197 genotyped	H	2,840	197	0.552	0.870	0.539	0.00	10.00	30.00	57.50
	P	18	197	0.857	0.348	0.870	60.00	60.00	40.00	70.00
	U	10,315	197	−0.583	1.310	−0.535	0.00	0.00	0.00	0.00
	H + P	2,858	197	0.771	0.763	0.770	60.00	50.00	40.00	60.00
	H + U	13,155	197	0.038	0.977	0.085	0.00	0.00	5.00	30.00
	P + U	10,333	197	0.801	0.434	0.792	40.00	30.00	35.00	60.00
	H + P + U	13,173	197	0.668	0.507	0.665	0.00	20.00	25.00	47.50
HBLUP 1203 ungenotyped	H	2,840	1,203	0.680	0.850	0.689	0.00	6.56	39.67	65.56
	P	18	1,203	0.943	0.151	0.945	67.74	55.74	62.81	80.50
	U	10,315	1,203	−0.701	1.406	−0.713	0.00	0.00	0.00	0.00
	H + P	2,858	1,203	0.892	0.685	0.873	67.74	55.74	57.02	78.84
	H + U	13,155	1,203	0.123	0.941	0.162	0.00	0.00	25.62	38.59
	P + U	10,333	1,203	0.965	0.060	0.950	67.74	55.74	62.81	80.50
	H + P + U	13,173	1,203	0.965	0.060	0.950	67.74	55.74	62.81	80.50
HBLUP 1,400 all	H	2,840	1,400	0.653	−1.952	0.660	20.00	15.71	35.71	62.50
	P	18	1,400	0.926	−0.065	0.929	42.86	50.00	61.43	75.36
	U	10,315	1,400	−0.683	2.206	−0.693	0.00	0.00	0.00	0.00
	H + P	2,858	1,400	0.868	−1.536	0.854	45.71	48.57	51.43	72.86
	H + U	13,155	1,400	0.116	0.767	0.157	2.86	11.43	14.29	34.64
	P + U	10,333	1,400	0.885	0.057	0.867	40.00	40.00	47.86	68.57
	H + P + U	13,173	1,400	0.907	0.021	0.893	40.00	45.71	54.29	72.14

*H = Hybrids; P = Parents; U = Uncles.

Table legend: Realized predictive abilities (RPA), bias of RPA, Spearman Rank correlation (EBVs × GEBVs/EGVs × GEGVs), and coincidence rates between the proportion of G_2_ candidate trees genomically selected at the seedling stage vs. phenotypically selected at age 6, under different selected proportions (%). NA: model did not converge. (Results for both ages 3 and 6 years, and all possible combinations of age adjustment models, genetic and genomic models, and training sets are provided in the **supplementary files**).

Genomic predictions using EBVs/EGVs estimated with a homogeneous versus a heterogenous variance model did not show any appreciable difference, but were slightly better when EBVs/EGVs were estimated using a homogeneous variance model (**Table S6**). Moreover, the RPAs obtained when HBLUP models were trained with the standard EBVs were largely equivalent and slightly better that the RPAs estimated when models were trained with de-regressed dEBVs. Taking as examples the RPAs obtained when models were trained with PARENTS, the RPAs were equal to 0.926 and 0.919 when EBVs and dEBVs were used as pseudo phenotypes, respectively. For the complete HYBRIDS+PARENTS+UNCLES training set, RPAs were 0.907 and 0.792 using EBVs and dEBVs to train models, respectively (**Table S7**).

Overall, higher RPAs were obtained as the G_1_ training set was more closely related to the G_2_ selection candidates in the HPT267, reaching values equal to or above 0.80 with the GBLUP_G and GBLUP_G+D models (**Table 2**). The most effective G_1_ training sets for the additive models were the ones containing the G_1_ PARENTS. Using the distantly related HYBRIDS for training resulted in lower additive RPAs (0.40 to 0.60), despite the 100× larger number of individuals involved in training (*n* = 1,842). The distantly related set of G_1_ individuals composed by the pure species UNCLES resulted in negative additive RPAs or non-convergence of the estimates. When combined with other training sets, UNCLES did not improve additive RPAs. However, when dominance was also predicted (GBLUP_G+D), PARENTS alone resulted in a low RPAs of 0.107, while the addition of HYBRIDS and UNCLES had a major positive impact on RPAs, increasing them 0.705 and 0.790 when HYBRIDS and UNCLES, respectively, were included in training (**Figure 2**; **Table 2**).

Overall, the improvement in RPA of HBLUP over GBLUP was marginal with only a slight increase of 2% from 0.539 to 0.552 when including ungenotyped HYBRIDS, and 3% from 0.775 to 0.801 when including ungenotyped UNCLES, while GBLUP trained with PARENTS alone was still better than HBLUP (**Table 2**). HBLUP predictions of the 1,203 ungenotyped individuals varied significantly depending on the training set used (**Table 2**) and no benefit was seen over ABLUP. The prediction bias for the additive models (GBLUP and HBLUP) indicated that RPAs were generally underestimated (1−*b*<1) when HYBRIDS alone were used, and overestimated (1−*b >*1) when UNCLES alone were used in training. RPA estimates using PARENTS were largely unbiased, with a value of 1−*b* close to zero (average of -0.111). The estimates of RPAs were underestimated for GBLUP_G+D for all training sets except when PARENTS alone were used. The lowest overall bias of RPA was seen when using HBLUP with PARENTS included in training (**Table 2**; **Table S5**).

### 3.4 Individual trees and family rank correlations between ABLUP and GBLUP/HBLUP

**Figure 3:**
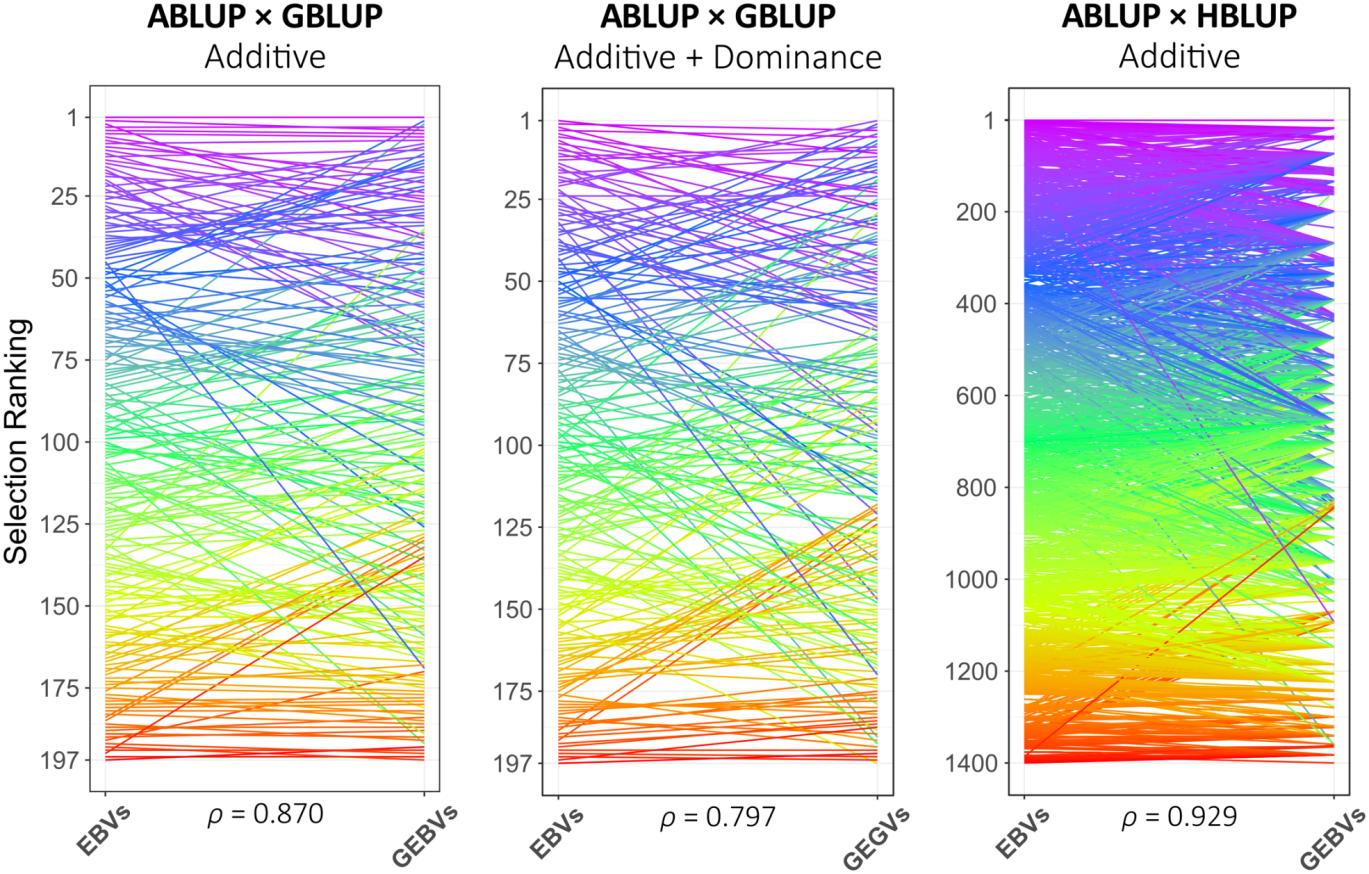
Comparative ranks of the G_2_ selection candidate trees in HPT267 based on ABLUP estimates of breeding values (EBVs) or genotypic values (EGVs) versus the ranks of the same trees based on their genomic counterparts (GEBVs or GEGVs), obtained with the best training set (see **Table 2**). Spearman’s rank correlations at the bottom of each panel.

EBVs for all 1,400 G2 trees in HPT267, GEBVs and GEGVs for the 197 genotyped trees, and GEBVs estimated with HBLUP for all 1,400 trees are provided for all analytical strategies implemented (**Table S8**). Spearman’s rank correlations for EBVs × GEBVs and EGVs × GEGVs for all analytical strategies were estimated (**Table S9**). Overall, rank correlations for additive values were close in value with the estimates of RPAs, showing the highest values for models trained using PARENTS (**Table 2**; **Table S9**). Rank correlations varied from 0.717 to 0.870 with GBLUP_G and reached similar values (0.694 to 0.797) with GBLUP_G+D only when HYBRIDS or UNCLES were added to PARENTS for training. Comparative plots of the phenotypic (ABLUP) vs. genomic (GBLUP or HBLUP) ranks are provided for the best-case training sets for each genomic model at age 6 (**Figure 3**), indicating trees from the top-ranked (purple) to the lowest-ranked (red) ones. Note that, in the ABLUP × HBLUP panel, the groups of ungenotyped individuals that belong to the same family were ranked genomically with the same GEBV value (lines converging to the same rank position in the HBLUP rank), in essence corresponding to ranking the families to which they belong.

The coincidence rates (%) between the number of G_2_ candidate trees in HPT267 that were genomically selected at the seedling stage and the number of trees that were ultimately selected based on phenotypes at age 6 are provided for all analytical strategies and selected proportions (**Table S10**), and summarized for age 6 under four selected proportions (2.5%, 5%, 10%, and 20%) (**Table 2**). For example, a selected proportion of 2.5% corresponds to selecting 5 trees in 197, and a 5% proportion corresponds to selecting 10 trees in 197. Coincidence rates at this small selected proportion (high selection intensity) were the highest for models trained with PARENTS using GBLUP_G or HBLUP (40 to 61% coincidence). In practice, this would amount to correctly selecting two or three individuals at the seedling stage based on DNA data, out of the five trees that would be ultimately selected based on actual growth measurement at age 6. Coincidence rates were much lower for the GBLUP_G+D model, only reaching 30% at 5% selected proportion, and 60% at 20% selected proportion. With selected proportions of 20%, coincidence rates above 70% were reached for GBLUP_G. Higher coincidence rates were reached with selected proportions above 20% (**Table S10**), although, in practice, breeders would hardly select more than 20% of the individuals in a hybrid progeny trial to be taken to a subsequent clonal trial. Coincidence rates using HBLUP of only the 1,203 ungenotyped individuals, corresponding in effect to family ranking, reached values of 67% at 2.5% and 80% at 20% selected proportions, indicating that GS at the family level would be highly efficient for early families’ screening (**Table 2**; **Table S10**).

**Figure 4:**
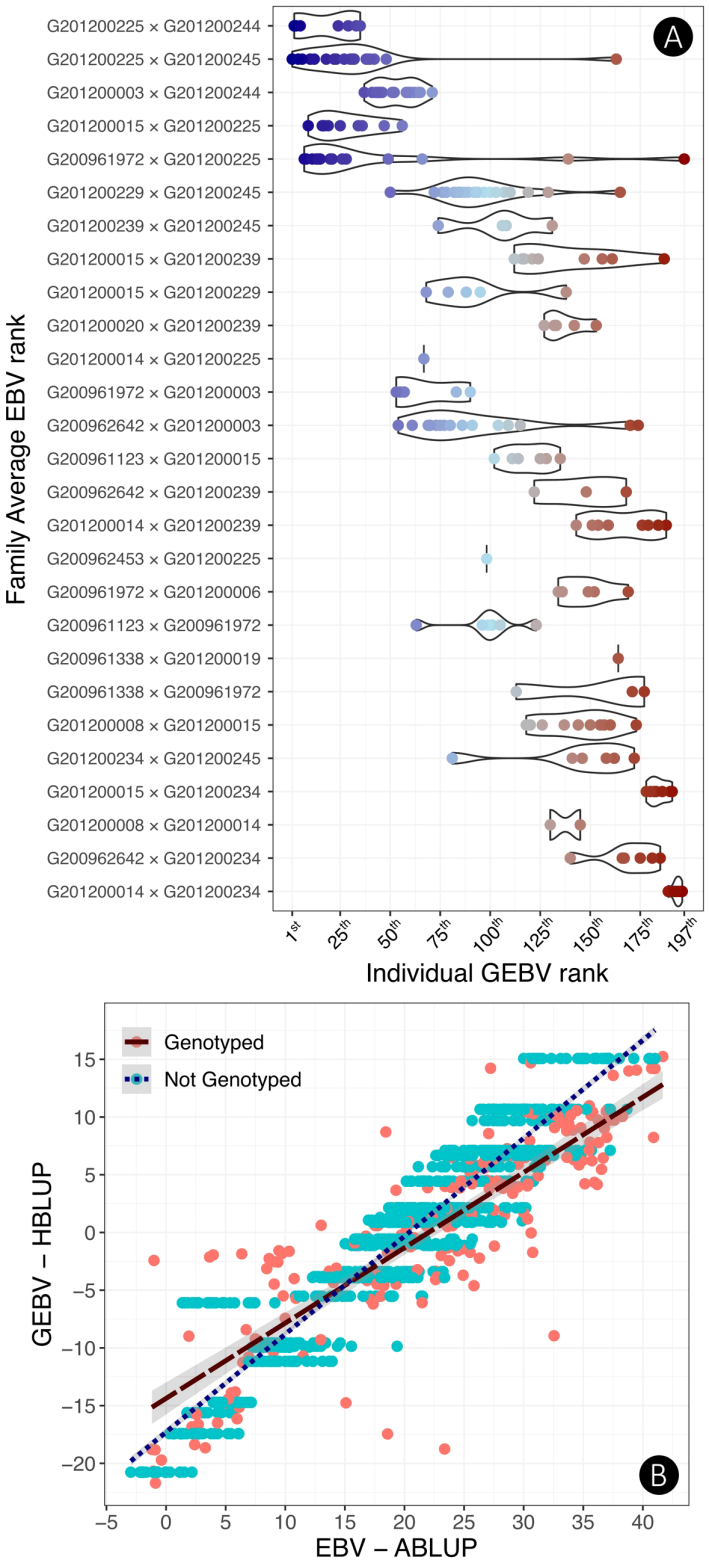
**(A)** Comparative rank of the full-sib families based on the family average EBV versus the individual GBLUP rank of the 197 genotyped selection candidates labeled by a color gradient going from the top-ranked trees (blue) to the bottom-ranked ones (red). **(B)** Comparative plot of the HBLUP GEBVs versus EBVs for all 1,400 trees in the progeny trial HPT267. Genotyped trees are labeled pink and ungenotyped trees are in blue. Ungenotyped selection candidates of the same full-sib family are ranked with the same GEBV as only pedigree information was available for them. Results presented were obtained with a Logistic 1 age adjustment at age 6, additive model trained with PARENTS.

The 197 genotyped individuals ranked by their GEBV were plotted according to the 27 unique families they belonged, and the 27 families, in turn, were ranked according to their average EBV (**Figure 4A**). The distribution of individuals shows a consistent pattern by which the top genomically ranked trees tended to belong to the top EBV ranked families with some occasional exceptions. For example, in the five top EBV ranked families that included 43 individuals, 25 trees were ranked among the top 30 GEBV ranked trees. **Figure 4B** shows an overall plot of EBVs vs. HBLUP estimated GEBVs for all 1,400 individuals, 197 genotyped (pink dots) and 1203 ungenotyped (blue dots).

### 3.5 Genomic predictions in the pure species breeding populations

**Figure 5:**
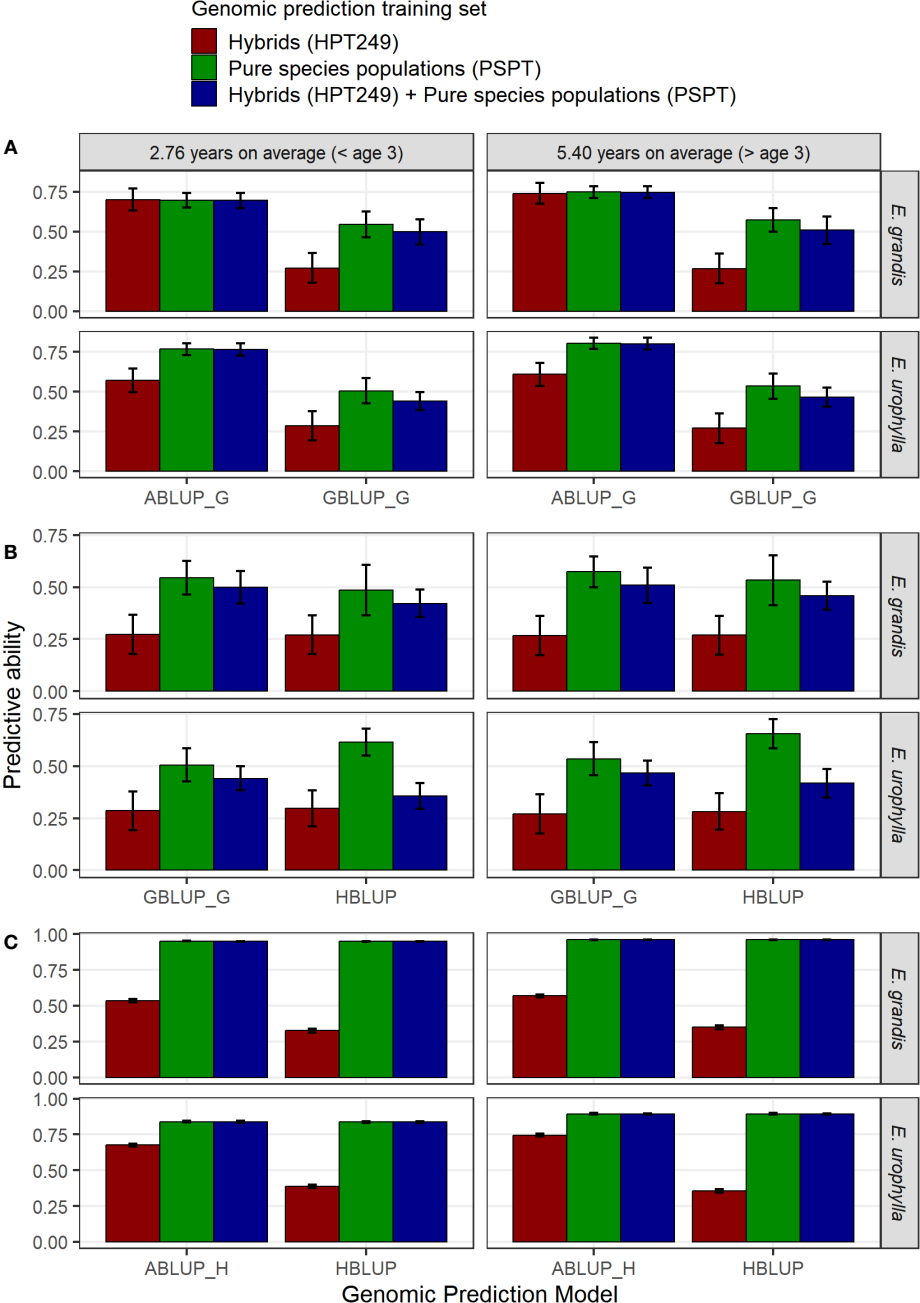
Comparison of genomic predictive abilities using different models and pure species and/or hybrid training sets to predict pure species individuals of *E*. *grandis* and *E*. *urophylla*. **(A)** ABLUP vs. GBLUP to predict genotyped but unphenotyped trees (ABLUP_G: only 197 genotyped individuals considered). **(B)** HBLUP vs. GBLUP to predict genotyped trees by including data of phenotyped but ungenotyped trees in training. **(C)** HBLUP vs. ABLUP to predict ungenotyped trees by including data of genotyped trees in training (ABLUP_H: all 1,400 individuals considered).

Results for both species, ages, and combinations of data adjustment and training sets are provided in **Table S11**, while results for the Logistic 1 adjustment are presented in **Figure 5**. When predicting MAI in the pure species, GBLUP and HBLUP cross-validated predictive abilities (PA) were generally twice as high when models were trained using exclusively each respective pure species data (PA ~0.50–0.75) compared to training using the hybrid data alone (PA ~0.20–0.30), while the pure species plus hybrid data combined provided intermediary values (PA ~ 0.40–0.50) (**Figure 5**). GBLUP was outperformed by standard ABLUP by ~50% when predicting unphenotyped trees (**Figure 5A**). HBLUP outperformed GBLUP to predict genotyped trees, improving the PA by 19%, from 0.51 to 0.61 at age 3, and by 22%, from 0.54 to 0.66 at age 6 when data of phenotyped but ungenotyped trees were included in training for *E. urophylla*. However, for *E. grandis*, the GBLUP model yielded slightly better PA (8% to 12%) than HBLUP (**Figure 5B**). Finally, there was no benefit in predictive ability of ungenotyped trees by including data of genotyped trees in training (**Figure 5C**).

## 4 Discussion

Most results of genomic prediction in forest trees to date have been derived from training and predicting breeding values based on cross-validation among individuals within the same breeding generation (Grattapaglia, 2022; Isik, 2022). In that approach, individual observations are randomly split into subsets, and all subsets except one are used as a training set with the remaining one serving as a validation population. Because the same population is both part of training and testing populations, relatedness between training and testing populations is maximized and no effects of recombination, selection, drift, and environmental changes are accounted for, often upwardly biasing predictive ability (Amer and Banos, 2010; Michel et al., 2016). In this study, however, we have provided experimental results of a forward GS across generations. In other words, we estimated not the “back-tested” retrospectively assessed predictive ability as previously reported (Bartholome et al., 2016; Isik et al., 2016; Haristoy et al., 2023), but rather the forward RPA for volume growth at harvest age. High realized predictive abilities were obtained at the individual and family mean level in an operational setting of a hybrid *Eucalyptus* RRS breeding program, corroborating earlier forecasts on the potential of GS in eucalypt breeding (Grattapaglia and Resende, 2011).

### 4.1 Data merging across trials and heritabilities

To evaluate the effectiveness of different training sets of the G_1_ generation, we consolidated phenotypic data across trials by age adjustment models. Age adjustments resulted only in slight improvement over unadjusted data. The fast growth of eucalypts in the tropics (Eldridge et al., 1993) and the strong age–age correlation for growth (Osorio et al., 2003; Bouvet et al., 2009a) likely explain this result. Nevertheless, the integration of phenotypic data from diverse trials, experimental settings, or breeding cycles requires establishing tree–age equivalencies and constitutes an invaluable approach for genomic prediction (Auinger et al., 2016). Furthermore, the incorporation of multiple datasets, from various geographical locations, provides the opportunity to explore environmental variation into enviromics approaches (Resende et al., 2021).

Besides consolidating phenotypic data across trials, the experiment required merging SNP genotypic data obtained at different times. Data portability across SNP genotyping platforms is a key aspect for the construction of legacy SNP databases for GS in long-lasting tree breeding programs. In Eucalyptus, the SNP arrays currently available (Silva-Junior et al., 2015) share large numbers of polymorphic SNPs, providing very high quality data for seamless data merging across time. This is not always the case, however, for other highly heterozygous tree species that still lack such resources. In those cases, SNP data are usually generated by genotyping-by-sequencing methods based on restriction enzyme-based complexity reduction, or sequence capture. With such methods, genotype reproducibility across sample batches is variable with considerable proportions of missing genotypes significantly complicating or even precluding data merging (Myles et al., 2010; Thistlethwaite et al., 2017; De Moraes et al., 2018; Darrier et al., 2019).

In our experimental setting, the estimates of heritability for MAI when estimated across all G_1_ trials combined (h_a_^2 = ^0.2 to 0.25) were similar to previous estimates in “urograndis” eucalypts (Bouvet and Vigneron, 1995; Vigneron and Bouvet, 2000; Van Den Berg et al., 2018). However, in the G_2_ target trial, heritability was higher (h_a_^2 = ^0.59), more in line with previous estimates in similar “urograndis” trials (Lima et al., 2019), likely due to better environmental control, which, in turn, contributed to the RPAs achieved. Models including the dominance effect with or without genomic data resulted in lower narrow-sense heritabilities, and broad-sense heritabilities higher than narrow-sense heritabilities. Genomic heritabilities followed the same pattern, and their estimates close to unity corroborate the fact that the recorded pedigree for the HPT267 trial was accurate and that the EBVs and EGVs used in our study are reliable baselines for the estimation of the RPAs (Klápště et al., 2018).

After including the dominance effect, the narrow-sense heritabilities decreased by 24%–27% at ages 3 and 6, respectively, while broad-sense heritabilities were higher than narrow-sense heritabilities. With HBLUP, heritabilities decreased by 56% at age 6 (**Table 1**). The dominance variance for MAI represented 39.5% and 36% of the additive variance and 28.3% and 26.5% of the total genetic variance at ages 3 and 6, respectively. Our results corroborate previous reports showing the relative importance of dominance to additivity in controlling volume growth in this hybrid (Bouvet et al., 2009b; Resende et al., 2017; Van Den Berg et al., 2018; Lima et al., 2019) and support the choice of the RRS strategy for breeding these superior hybrids. Moreover, the considerable reduction in narrow-sense heritability substantiates the fact that the pedigree-based analysis cannot capture the complete genetic relationship among individuals, failing to unscramble the non-additive genetic component from the additive one (Munoz et al., 2014).

### 4.2 EBVs versus de-regressed EBVs as pseudo phenotypes in GBLUP

In our study, we evaluated whether using de-regressed EBVs (dEBVs) or standard EBVs as pseudo phenotypes to train prediction models would result in any appreciable difference in genomic predictions. De-regression of EBVs (Garrick et al., 2009) was proposed as a BLUP correction before genomic model fitting to deal with a possible covariance between residuals, containing a genetic part not captured by the EBVs ( $\hat{g}\;-\;g$ ), and the true breeding value ( g ) in a genomic model. De-regressed EBVs have frequently been used in GS studies in forest trees (Resende et al., 2012a; Isik et al., 2016; Thistlethwaite et al., 2019; Haristoy et al., 2023) as a way to improve GS accuracy by taking into account the heterogeneous reliability of EBVs, due to the unbalanced nature of forest trial data. In our study, however, EBVs and dEBVs were essentially the same and genomic models trained with EBVs were largely equivalent or slightly better than those trained with dEBVs. These results are in line with previous reports in forest trees that showed high correlations (>0.93) between EBVs and dEBVs for growth data in loblolly pine (Resende et al., 2012b), or no effect of de-regression on genomic predictions (Bartholome et al., 2016; Isik et al., 2016; Haristoy et al., 2023).

It is important to point out that Garrick´s method was proposed as a way to mitigate a common issue of genomic prediction in animal breeding where training set data might involve genotyped animals with alternative types of information including single or repeated measures of individual performance, information on progeny estimated breeding values, or a pooled mixture of more than one of these information sources. The reliability of such measures likely varies and hence the method finds relevant application. However, as Garrick points out at the end of that famous paper, he concludes that: “*In practice, the benefit of deregression and the subsequent weighting of alternative information sources will depend on the extent to which the number of repeat records, number of progeny and/or r^2^ varies among individuals in the training population”* (Garrick et al., 2009). Therefore, in forest tree trials where frequently families are the selection units (Isik et al., 2016), many family members are evaluated and clonal replicates are available as experimental units (Haristoy et al., 2023) or clonal checks, like in our study, reliabilities are usually high and the advantage of de-regression not necessarily happens.

### 4.3 Model training for genomic prediction of hybrids in RRS

Whereas the general approach to training a GS model in a recurrent selection breeding strategy is well settled (Massman et al., 2013), this is not so for reciprocal recurrent GS. It is not yet clear whether a GS model should be trained on the pure species or the hybrid crossbred data of prior generations for selecting individuals in terminal hybrid crosses. In domestic animal breeding, reports vary depending on the species, trait, and breeds involved. While breed-specific effects warrant training genomic models on crossbred data in pigs (Lopes et al., 2017) and chickens (Duenk et al., 2019), a review in bovines shows that the advantages of breed-specific allelic effects are small, and training on pure species data for additive variance would be more effective (Mcewin et al., 2021). In forest trees, to the best of our knowledge, our study provides the first experimental data on this issue. In our experimental setting, training only with the G_1_ HYBRIDS generation allowed a still acceptable additive RPA of approximately 0.60 in the subsequent generation of hybrids. Predictions were largely improved when models were trained with the pure species parents combined. It is true, however, that the HYBRIDS training set, although 100× larger, was related to the PARENTS only as hybrid half-sibs and therefore more distant from the G_2_ selection candidates that impacted the predictions considerably (**Figure 1**). Interestingly, however, GBLUP_G+D models trained exclusively with the pure species data of PARENTS resulted in much lower RPAs when compared to GBLUP_G (**Figure 2**; **Table 2**). Offspring share only one copy identical by descent (IBD) inherited from their parents. Because dominance effects are based on sharing two copies, GEGVs of offspring by principle cannot be estimated from training with the pure species parental data alone. Accordingly, the inclusion of hybrid data or pure species relatives to predict non-additive genetic effects by GBLUP_G+D was essential to attain maximal RPAs close to 0.80 (**Figure 2**; **Table 2**).

Results with the HBLUP model in the HPT267 showed that the inclusion of phenotype data of a large number of ungenotyped G_1_ trees only marginally improved the RPA beyond the contribution of the genotyped trees in the training set, in contrast to the positive results of HBLUP reported in other forest tree studies (Ratcliffe et al., 2017; Cappa et al., 2019; Callister et al., 2021; Quezada et al., 2022; Walker et al., 2022). Two reasons might account for this result: (1) the distant genetic relationship between the added ungenotyped UNCLES individuals and the hybrid selection candidates, and (2) the overwhelming genetic contribution to the prediction model of the closely related PARENTS to the selection candidates. Our results confirm several previous results in forest trees showing that higher relatedness between training and selection populations improves genomic prediction (reviewed in Grattapaglia et al., 2018; Isik, 2022). The crucial role of relatedness in GS was shown early on in the first experimental study of GS in *Eucalyptus* when prediction across two unrelated breeding populations failed (Resende et al., 2012a). This same observation was followed by several studies in all mainstream forest tree species, compiled in a recent statistical analysis of GS studies, where the effect of relatedness was shown to be highly significant on prediction accuracy (Isik, 2022). Close relationship increases the probability that chromosome segments IBD sampled in the training set are also found in the selection candidates (Daetwyler et al., 2013).

The high RPAs above 0.80 and up to 0.90 (**Table 2**) obtained with the additive models trained exclusively with the 18 parents of the G_2_ generation could be somewhat surprising when considering previous simulations showing that large training populations would be necessary to attain satisfactory predictive abilities (Grattapaglia and Resende, 2011). Nevertheless, two cross-generational studies in conifer trees that also evaluated the exclusive use of the parents of selection candidates to train a prediction model for additive effects showed equivalent results. In *Pinus pinaster*, high predictive abilities for additive effects of growth were high (0.70 to 0.91) using exclusively the 46 G_0_ and 62 G_1_ parents to predict G_2_ individuals (Bartholome et al., 2016). In *Pseudotsuga menziesii*, models trained only with the F_1_ generation parents predicted F_2_ individuals with a predictive ability of 0.92 (Thistlethwaite et al., 2019), when parental average effects were not removed. The longer extensions of shared haplotypes resulting from co-segregation and the direct parent–offspring relatedness, with only a single preceding recombination, undoubtedly were the drivers of the high RPAs observed in those studies as well as ours. Longer shared haplotypes have been shown to be more important in determining the predictive ability than the isolated length of shared haplotypes (Wientjes et al., 2013). While our report seems to indicate that training a model only with the direct parents of selection candidates is sufficient to train an effective GS model, this might be specific to this eucalypt hybrid situation, and further data in different experimental settings should be gathered to validate such an approach.

From the practical standpoint, the fundamental expectation of GS is that a prediction model should be useful for several sequential breeding generations, although a decline in predictive ability is expected due to recombination and selection along the breeding generations, as pedigree relationships and occasional marker-QTL LD dissipates (Meuwissen et al., 2001; Long et al., 2011; Müller et al., 2017b). Therefore, it would be impractical to count on a sustained direct parent–offspring relationship for predictions. Continuous model updating with new data can, however, be adopted to continuously maintain training-to-candidates genetic relationships as close as possible (Iwata et al., 2011). In fast-growing tropical eucalypts, it is logistically possible to grow and measure a subset of the genotyped seedlings of every breeding generation to harvest age (Grattapaglia, 2014). These new training data substitute older generation data by updating the prediction model, thus providing persistent predictive abilities.

### 4.4 GS allows accurate individual tree ranking

GS in forest trees has been traditionally formulated as a problem of predicting the breeding value of an individual to be used as a parent in the subsequent generation. However, in the case of RRS, besides accelerating the progress of the pure-species populations, GS is intended for early selection of individual trees in terminal hybrid trials. The top-ranked selected hybrids are vegetatively propagated and taken to clonal trials. Because clonal propagation exploits both additive and non-additive effects, both have to be predicted into the tree’s GEGV. GS therefore becomes more of a problem of individual tree ranking for total genotypic value as pointed out earlier (Blondel et al., 2015) and needs a different validation scheme (Daetwyler et al., 2013). We therefore estimated a rank correlation between the pedigree and genomic estimated genetic values. In our experiment, individual tree ranks predicted by genomic data, both by GEBVs or GEGVs, closely followed the ranks based on their EBVs or EGVs. Rank correlations ≥0.80 were estimated using the most effective training sets (**Figure 3**; **Table 2**). Coincidence rates of the top-ranked individuals were highest for GBLUP_G and HBLUP models reaching 60% at the highest selection intensity (smallest selected proportion of 2.5%). However, when dominance effects were included, despite an overall satisfactory overall rank correlation of 0.797, the GEGVs rank deviated from the EGVs rank at the granular level, only reaching reasonable coincidences of 35% at selected proportions of 10% and above. In other words, GBLUP_G+D did not identify the exact same individuals that would be selected based on their EGVs at the extreme of the rank distribution, although with higher selected proportions coincidence improved.

These results indicate that while GS allows successful prediction and ranking of individuals within families by their GEBVs, ranking by GEGV will be more challenging. In comparing ranks of estimated total genotypic values, it is important to recall, however, that both EGV and GEGV are only modeled attempts to estimate the true total genotypic value, which is actually unknown (Falconer, 1989; Lynch and Walsh, 1998). Although the EGVs were calculated from the trees’ observed performances, rank position at the extremes of the distribution might be impacted by additional non-additive variation unaccounted by the model. In fact, it has been reported that the correlation of growth performance of a eucalypt “urograndis” hybrid ortet and its clonal ramets is typically poor (Furtini et al., 2012; Van Den Berg et al., 2015). Only a replicated field clonal trial of a more generous selected proportion of top GEGV ranked individuals might ultimately capture the top EGVs ranked trees.

### 4.5 Genomic selection and ranking of families and individuals in a two-stage GS approach

We have shown that the genomic data accurately predict and rank families by the average genomic breeding value, closely matching the rank by their average EBVs (**Figure 5**). Furthermore, the results show that the top-ranked full-sib families contain the majority of the top-ranked individual trees. These empirical results have two practical implications. First, they support earlier suggestions of using GS when the selection unit for commercial deployment is elite families and not individual trees (Resende et al., 2017). Top genomically predicted families could be produced by large-scale mating between top parents, and additionally scaled up by clonal propagation in a family forestry (White et al., 2007) or clonal composite (Rezende et al., 2019) deployment strategies. The second consequence is that the accurate family ranking obtained in our study empirically corroborates a recent proposition of a two-stage GS approach (Grattapaglia, 2022). In the first stage, a larger-than-usual number of full-sib families would be produced, a small sample of seedlings genotyped per family either individually (Rios et al., 2021) or in DNA pools (Cericola et al., 2018), and the data used to rank the families by their average GEBV. For the top-ranked families, a larger number of individual seedlings would be genotyped, and by accounting for the Mendelian segregation, they would be ranked by their GEBV or GEGV. This GS approach would radically increase both between- and within-family selection intensity while significantly optimizing genotyping costs.

The proposed two-stage GS strategy fits exceptionally well with a cloned progeny trial approach (Chen et al., 2020), a current trend in advanced eucalypt hybrid breeding, as a way to improve the correlation between the growth performance of an individual tree in a progeny trial and its clone in a clonal trial. A cloned progeny trial provides increased accuracy for individual selection by boosting individual tree heritability, and cuts in time by consolidating progeny and clonal trial in one step. However, it requires significant logistic effort and it is therefore constrained by the number of clones that can be reasonably tested, thus reducing selection intensity when compared to the conventional two-step progeny followed by the clonal trial. The two-stage GS scheme (see **Figure 2** in Grattapaglia, 2022) provides an elegant solution to this limitation with a small genotyping effort to genomically rank families and devoting larger genotyping effort to the offspring individuals of the pre-selected families that offer the highest probability of ultimately delivering top clones. The experimental data gathered in this study show that such an approach would have promptly identified the top five families that contain almost all 50 top GEBV ranked individual trees (**Figure 4**).

### 4.6 Reciprocal recurrent GS for faster advancement of pure species breeding populations

We have shown the importance of including hybrid data for predicting non-additive effects in hybrid selection candidates. However, would prior generation hybrid data be necessary or useful for genomic predictions of pure species individuals? Results showed that hybrid data not only did not provide acceptable predictive abilities by itself, but also did not improve predictions when added to the pure species data. Pure species data alone were effective for training satisfactory genomic models (**Figure 5**). This result is relevant in the context of the long time needed to advance an RRS program, still considered a limitation for a more widespread use of this strategy for “urograndis” hybrids. GS integrates well into the alternative RRS strategy selecting forward (RRS-SF) (Nikles, 1992). RRS-SF was proposed several years ago with the specific aim to shorten the conventional RRS by omitting the backward selection step while producing and testing pure species and hybrids simultaneously in each generation. Hybrid and pure species performance data are analyzed simultaneously, combining parental GCA information for pure species and for hybrid performance with individual-tree data to get EBVs for forward selection of pure species progeny (Kerr et al., 2004). Our pure species HBLUP models adopted this approach, but showing that the hybrid data did not contribute appreciably to the predictive abilities of the pure species. This result agrees with previous reports showing that individual breeding values of *E. urophylla* pure species trees were good indicators of their parent performance as hybrid partners with *E. grandis* for tree volume (Van Den Berg et al., 2015).

In cross-validation, ABLUP significantly outperformed GBLUP (**Figure 5A**) with all training sets, a common result seen in eucalypts (Müller et al., 2017a; Tan et al., 2018; Cappa et al., 2019) and other forest trees (Munoz et al., 2014; Gamal El-Dien et al., 2016). Inflated predictive abilities have been attributed to the inability of the ABLUP model to unscramble the significant non-additive variance component for volume growth. Moreover, we did not see any benefit in including data of genotyped trees in training by HBLUP, on the predictive ability of ungenotyped trees. Most likely, the added information of only ~100 genotyped individuals was insufficient to propagate to the ~5,000 ungenotyped trees to provide any relevant genetic relationship connections for this particular HBLUP application. However, the inclusion of phenotype data of ungenotyped individuals in the HBLUP models resulted in a ~20% improvement of genomic predictions over standard GBLUP for *E. urophylla.* These results corroborate previous reports on the value of HBLUP over GBLUP in boosting genomic predictions in *Eucalyptus* (Cappa et al., 2019; Callister et al., 2021; Quezada et al., 2022). However, for *E. grandis*, the predictive abilities with HBLUP were slightly worse than with GBLUP. Notwithstanding potential pedigree inconsistencies in the *E. grandis* PSPTs, fewer individuals were genotyped for this species, and the average relationship between genotyped and ungenotyped trees was slightly lower (0.17) than in *E. urophylla* (0.21) (data not shown). These results highlight the fact that the expected advantage of HBLUP relies heavily on precise pedigree records and the level of relationship between the genotyped and ungenotyped individuals (Klápště et al., 2018).

## 5 Concluding remarks and perspectives

We have carried out the first forward GS experiment across generations in eucalypts, also unprecedented in what concerns reciprocal recurrent GS in forest trees. Our results add to a recent back-tested GS study across three generations of *E. globulus* breeding, also showing very encouraging results (Haristoy et al., 2023). Our realized predictive abilities for additive, and additive plus dominance effects reached or exceeded 0.8. Individual tree GEBV or GEGV and family GEBV ranks closely matched their pedigree-based counterparts, with rank correlations also above 0.80. Our results further validated the general consensus in the practice of GS irrespective of species: higher relatedness between training and selection populations improves predictions (Ahmadi and Bartholomé, 2022). Our best training sets not only were just one generation apart from the selection candidates, but also were their direct parents. This result suggests that a modest genotyping effort of a small training set strongly related to the selection candidates could be sufficient to carry out GS. Training genomic models on EBVs and fully exploiting the longer extensions of shared haplotypes between parents and offspring will maximize additive predictions, while prediction of the dominance component requires training on individuals sharing genotypes with selection candidates. We recognize, however, that more experimental data are necessary to assess the practicality of sustaining such direct relatedness in different breeding programs as generations advance.

Traditionally, expectations have been that long-term and wider interpopulation genomic prediction will rely not only on relatedness but also on linkage disequilibrium (LD) information, although the distinction between these two components is somewhat subjective (Daetwyler et al., 2013). In practice, however, experimental results to date in forest trees have failed to capture what is called “true LD”, whatever that means, even when using relatively dense genotyping in the much smaller eucalypt genome when compared to conifers (Müller et al., 2017a; Resende et al., 2017). This has led to suggestions that simply tracking relatedness with DNA markers without capturing LD would be of little value over conventional ABLUP for forward within-family selection in forest trees, and that the ultimate aim of using GS should be to capture true LD across populations (Thistlethwaite et al., 2019). Our view, however, is that the value of GS should be assessed from a practical perspective of the improved genetic gain per unit time versus cost of implementation in the specific population relevant to each breeding program. Predicting complex traits widely across unrelated populations of different breeding programs based on LD, notwithstanding its improbability, might therefore not even be a relevant goal in the current landscape of industrially oriented advanced tree breeding.

At least in industrial eucalypt improvement, GS will be practiced within specific breeding populations with effective sizes rarely exceeding *N_e_* = 50, and genomic predictions will be carried out across one or at most two generations before model updating is done. Furthermore, both components of an individual’s breeding value are very relevant: the parent average breeding value and the within-family Mendelian term due to the sampling of gametes from its parents, the latter accounting for 50% of interindividual genetic differences in breeding values. Additionally, the non-additive variance is also typically captured by clonal propagation. Thus, marker-based prediction of differences among full-sibs due to Mendelian sampling is very important in achieving genetic gain. While pedigree-based BLUP predictions can yield accurate estimates of parental average when ancestors data are abundant, Mendelian segregation terms require records from progeny. As we have shown in this study, genomic data can accurately predict additive Mendelian sampling without progeny records, enabling ultra-early and accurate selective ranking at the seedling stage, although less efficiently when the total genotypic value was predicted, at least in this experiment. Moreover, the granular estimates of relatedness within families provided by genomic data will also allow much better management of inbreeding and maintenance of Mendelian segregation variance for continued gains when compared to pedigree alone (Daetwyler et al., 2007; Nirea et al., 2012).

Finally, besides providing experimental data on cross-generational GS in the hybrid terminal trials, our study also provided indications that training models with pure species data should yield high predictive abilities that can be boosted by an HBLUP approach to accelerate the progress of the reciprocal populations in RRS breeding. Realized predictive abilities across generations for the pure species populations will await ongoing experimental work, although we speculate that similarly positive results are likely to emerge. As GS in forest tree breeding is now “climbing the slope of enlightenment” as a workable breeding “technology” (Grattapaglia, 2022), we expect that an increasing number of experimental reports of forward GS across generations will get published, and innovative optimization of genotyping costs will happen, ultimately driving the adoption of GS by the tree breeders’ community worldwide.

## Data availability statement

The datasets presented in this study can be found in the online repository figshare.com with the following D.O.I.: 10.6084/m9.figshare.23582370. Full results of data analyses presented in the study are included in the **Supplementary Material**. Further inquiries can be directed to the corresponding author.

## Author contributions

DG, ET, and RR contributed to the conception and design of the study. ET and JS performed the field experimental work. GS carried out data analysis with feedback from RR. DG wrote the manuscript and all co-authors subsequently contributed to it by editing the final version. All authors contributed to the article and approved the submitted version.

## Supplementary material

File S1 Details of the linearization of the age adjustment models.

Supplementary Figure 1 Linear and non-linear models fitting for four experimental units (trees) used to equalize the age differences between them to two time points at 2.76 and 5.40 years.

Supplementary Table 1 Narrow sense (h_a_^2^) and broad sense (h_g_^2^) heritabilities and variance components for mean annual increment (MAI) in tree volume estimated by ABLUP for all progeny trials (G_1_ and G_2_ generations) combined at the two ages with the different combinations of age and genetic adjustment models.

Supplementary Table 2 Narrow sense (h_a_^2^) and broad sense (h_g_^2^) genomic heritabilities and variance components for mean annual increment (MAI) in tree volume at age six estimated in the HPT267 with the different combinations of age adjusted data, genetic and genomic models (*Model did not converge).

Supplementary Table 3 Correlations between EBVs estimated with a homogenous versus a heterogenous residual variances model. Correlations are listed for ages three and six, different age and genetic model adjustments, all G1 and G2 trials and both genetic models (A and A+D). (Obs. the A+D model with heterogenous variances did not converge for the age adjusted data).

Supplementary Table 4 Correlations between the EBVs and de-regressed dEBVs of the individuals used in each combination of training set and genomic model. Estimates are provided for all age adjustment and genetic models at ages 3 and 6.

Supplementary Table 5 Realized predictive abilities (RPAs) in the G2 generation (HPT267) for mean annual increment (MAI) in tree volume and corresponding estimates of bias at the two measurement ages estimated with the different combinations of age adjustment, genetic and genomic models, using different G1 generation training sets.

Supplementary Table 6 Comparison of genomic predictive abilities (RPAs) using the HBLUP model with EBVs estimated under a homogeneous or heterogeneous variances model for the cases of unadjusted and Logistic 1 age adjusted and all training sets. Shown are the correlations of GEBVs and the respective RPAs and their differences at age six years.

Supplementary Table 7 Comparison between the realized predictive abilities (RPA) estimated using Logistic 1 age adjusted data, additive model at age 6, EBVs and de-regressed dEBVs with HBLUP model for the different training sets.

Supplementary Table 8 EBVs (Estimated Breeding Values), EGVs (Estimated Genotypic Values), GEBV (Genomic Estimated Breeding Values) and GEGV (Genomic Estimated Genotypic Values) for mean annual increment (MAI) in tree volume of the 1400 individual trees of HPT267 out of which 197 were genotyped. Estimates were obtained using different combinations of age adjustment, genetic model and genomic models and training sets.

Supplementary Table 9 Spearman Rank correlations between the EBVs (Estimated Breeding Values) x GEBV (Genomic Estimated Breeding Values) or EGVs (Estimated Genotypic Values) x GEGV (Genomic Estimated Genotypic Values). Correlations are listed for the different combinations of age adjustment, genetic and genomic models, for the different training sets.

Supplementary Table 10 Coincidence rate (%) between the number of G2 candidate trees in HPT267 that would be selected based on their GEBV (Genomic Estimated Breeding Values) or GEGV (Genomic Estimated Genotypic Values) and the number of trees that were selected based on their EBVs (Estimated Breeding Values) or EGVs (Estimated Genotypic Values) at age six for different selected proportions (%). Correlations are listed for the different combinations of age adjustment, genetic and genomic models, for the different training sets.

Supplementary Table 11 Predictive abilities (PAs) for mean annual increment (MAI) in tree volume and corresponding standard errors estimated by cross-validation (Pure species and Pure species + Hybrids training sets) and standard errors of Pearson correlation (Hybrids training set) for each pure species breeding population in the RRS program. Estimates listed for the two measurement ages with different combinations of age correction, genetic and genomic models, using different G_1_ generation training sets. NA: model did not converge.
