# Supplementary material for: Realized genomic selection across generations in a reciprocal recurrent selection breeding program of Eucalyptus hybrids
Source: Front Plant Sci. 2023 Oct 27;14:1252504. doi: 10.3389/fpls.2023.1252504 (PMC10641691; doi:10.3389/fpls.2023.1252504)
Supplement: File S1 — Details of the linearization of the age adjustment models. [file DataSheet_1.pdf]

Supplementary File S1. Linearization of the age correction models

The linearization of the non-linear **Logistic 1 model** was given by:

$$ln\left(\frac{1}{y_i} - 1\right) = ln\left(\beta_{0_i}\right) + \beta_{1_i}x$$

Where y_i is the observation of the i-th individual tree of a given genotype, experiment and block; β_{0_i} e β_{1_i} are the parameters of the respective models; x is the age. Thus, the terms $ln\left(\beta_{0_i}\right)$ and β_{1_i}, correspond to the intersection parameters of the line on the vertical axis (β'_{0_i}) and the linear regression coefficient (β'_{1_i}), respectively. This linear model was adjusted and, subsequently, the value of y_i was estimated by:

$$y_i = \frac{1}{1 + e^{\left[\beta'_{0_i} + \beta'_{1_i}x\right]}}$$

The linearization of the non-linear **Logistic 2 model** was given by:

$$ln\left(\frac{1}{y_i} - 1\right) = -\beta_{1_i}\, ln\left(\beta_{0_i}\right) + \beta_{1_i}\, ln(x)$$

Thus, the terms $-\beta_{1_i}\, ln(k_i)$ e β_{1_i} correspond to the intersection parameters of the line on the vertical axis (β'_{0_i}) and the linear regression coefficient (β'_{1_i}), respectively. This linear model was adjusted and, subsequently, the value of y_i was estimated by:

$$y_i = \frac{1}{1 + e^{\left[\beta'_{0_i} + \beta'_{1_i} ln(x)\right]}}$$

The linearization of the **Gompertz model** was given by:

$$ln\left[ln\left(\frac{1}{y_i}\right)\right] = \beta_{0_i} - \beta_{1_i}x$$

Thus, the terms β_{0_i} e $-\beta_{1_i}$, correspond to the intersection parameters of the line on the vertical axis (β'_{0_i}) and the linear regression coefficient (β'_{1_i}), respectively. This linear model was adjusted and, subsequently, the value of y_i was estimated by:

$$y_i = e^{-e^{\beta'_{0_i} + \beta'_{1_i}x}}$$
