# Supplementary material for: Realized genomic selection across generations in a reciprocal recurrent selection breeding program of Eucalyptus hybrids
Source: Front Plant Sci. 2023 Oct 27;14:1252504. doi: 10.3389/fpls.2023.1252504 (PMC10641691; doi:10.3389/fpls.2023.1252504)
Supplement: Supplementary Figure 1 — Linear and non-linear models fitting for four experimental units (trees) used to equalize the age differences between them to two time points at 2.76 and 5.40 years. [file DataSheet_2.pdf]

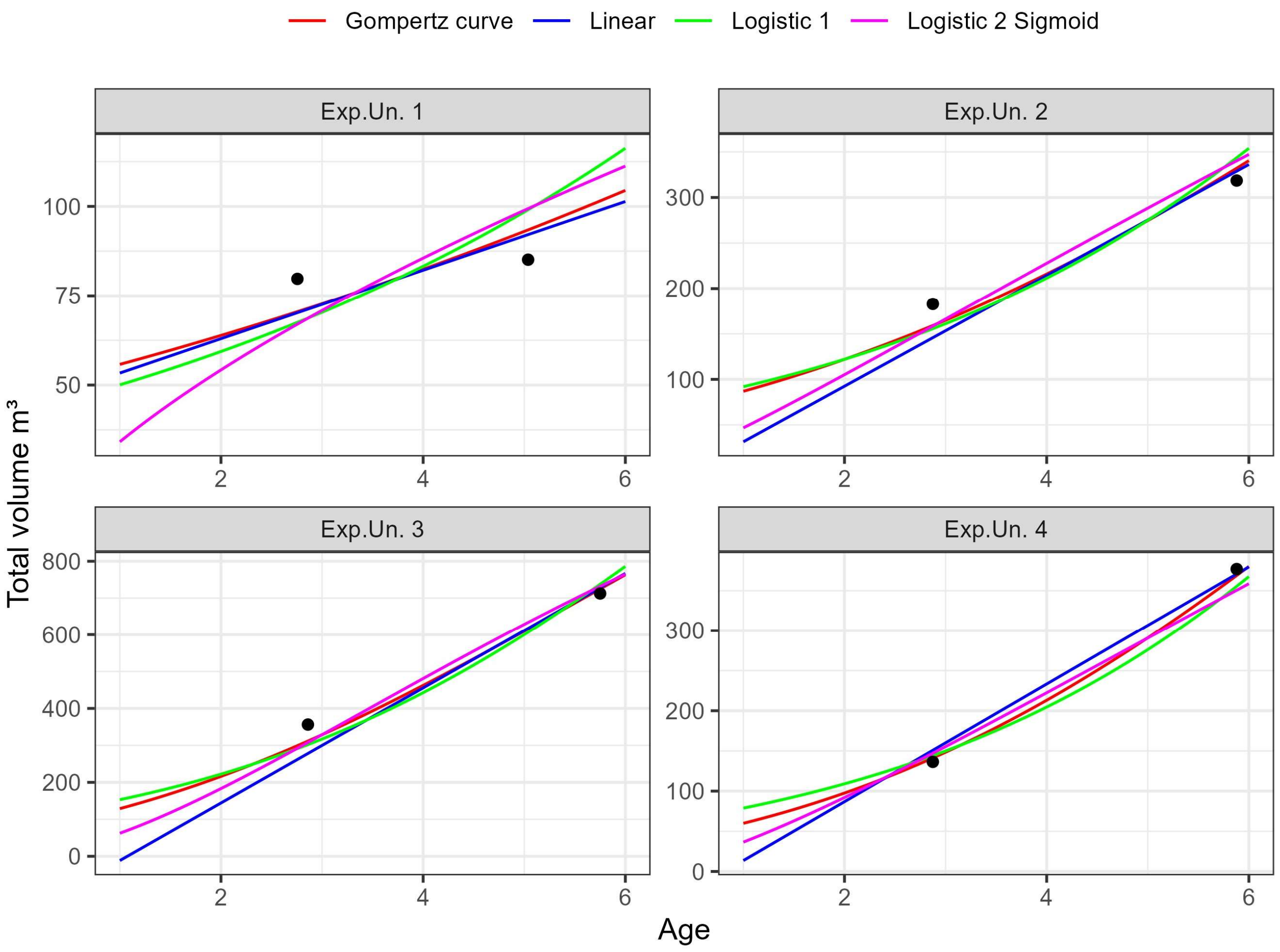

Supplementary Figure S1. Linear and non-linear models fitting for four experimental units (trees) used to equalize the age differences between them to two time points at 2.76 and 5.40 years.
